# *Echinococcus* metacestode: in search of viability markers[Fn FN1]

**DOI:** 10.1051/parasite/2014063

**Published:** 2014-11-28

**Authors:** Bruno Gottstein, Junhua Wang, Oleg Blagosklonov, Frédéric Grenouillet, Laurence Millon, Dominique A. Vuitton, Norbert Müller

**Affiliations:** 1 Institute of Parasitology, Vetsuisse Faculty and Faculty of Medicine, University of Bern Switzerland; 2 Department of Nuclear Medicine, University of Franche-Comté and Jean Minjoz University Hospital Besançon Franche-Comté France; 3 Laboratory of Parasitology-Mycology, Centre Hospitalier Universitaire, Université de Franche Comté Besançon France; 4 WHO-Collaborating Centre for the Prevention and Treatment of Human Echinococcosis, University of Franche-Comté and University Hospital Besançon Franche-Comté France

**Keywords:** *Echinococcus multilocularis*, Alveolar echinococcosis, Surgery, Medication, Follow-up, Em18, Em2, FDG-PET

## Abstract

Epidemiological studies have demonstrated that most humans infected with *Echinococcus* spp. exhibit resistance to disease. When infection leads to disease, the parasite is partially controlled by host immunity: in case of immunocompetence, the normal alveolar echinococcosis (AE) or cystic echinococcosis (CE) situation, the metacestode grows slowly, and first clinical signs appear years after infection; in case of impaired immunity (AIDS; other immunodeficiencies), uncontrolled proliferation of the metacestode leads to rapidly progressing disease. Assessing *Echinococcus multilocularis* viability *in vivo* following therapeutic interventions in AE patients may be of tremendous benefit when compared with the invasive procedures used to perform biopsies. Current options are F18-fluorodeoxyglucose-positron emission tomography (FDG-PET), which visualizes periparasitic inflammation due to the metabolic activity of the metacestode, and measurement of antibodies against recEm18, a viability-associated protein, that rapidly regresses upon metacestode inactivation. For *Echinococcus granulosus*, similar prognosis-associated follow-up parameters are still lacking but a few candidates may be listed. Other possible markers include functional and diffusion-weighted Magnetic Resonance Imaging (MRI), and measurement of products from the parasite (circulating antigens or DNA), and from the host (inflammation markers, cytokines, or chemokines). Even though some of them have been promising in pilot studies, none has been properly validated in an appropriate number of patients until now to be recommended for further use in clinical settings. There is therefore still a need to develop reliable tools for improved viability assessment to provide the sufficient information needed to reliably withdraw anti-parasite benzimidazole chemotherapy, and a basis for the development of new alternative therapeutic tools.

## Abstract (fr)

Les études épidémiologiques suggèrent que la plupart des personnes infectées par les métacestodes d’échinocoques sont résistantes au développement d’une maladie. Chez les malades, la croissance parasitaire est partiellement contrôlée par l’immunité de l’hôte : s’ils sont immunocompétents, un développement lent du métacestode est à l’origine de l’échinococcose alvéolaire (EA) ou kystique (EK) et les premiers signes apparaissent des années après l’infection; s’ils sont immunodéprimés (sida, autres situations d’immunosuppression), une prolifération incontrôlée du métacestode s’accompagne d’une évolution rapide. Évaluer *in vivo* la viabilité larvaire après intervention thérapeutique chez les patients atteints d’EA représenterait un avantage considérable sur les méthodes invasives (biopsies des lésions). Les options non-invasives sont la tomographie par émission de positons au F-18 fluorodeoxyglucose (FDG-TEP), qui visualise l’inflammation péri-parasitaire associée à l’activité métabolique du métacestode, et la détermination des taux d’anticorps dirigés contre Em18, une protéine associée à la viabilité parasitaire; leur sécrétion régresse rapidement lors de l’inactivation du métacestode. Pour *E. granulosus*, on manque de paramètres similaires; on peut cependant lister quelques candidats. D’autres marqueurs sont à l’étude : ils incluent l’imagerie en résonance magnétique fonctionnelle ou pondérée en diffusion, et le dosage de substances parasitaire (antigènes circulants, ADN), ou sécrétées par l’hôte (marqueurs d’inflammation, cytokines, chimiokines). Bien que certains aient paru prometteurs dans des études pilotes, aucun n’a été validé sur un nombre suffisant de patients pour pouvoir être recommandé en suivi clinique en routine. Il reste indispensable de développer des outils fiables susceptibles d’apporter l’information nécessaire pour arrêter la chimiothérapie par benzimidazoles, ou d’être utilisés pour le développement de nouveaux outils thérapeutiques.

## 1. Introduction

The genus *Echinococcus* includes more than seven different species plus multiple genotypes [38]. Among them, *Echinococcus multilocularis* (the small fox tapeworm) is the most pathogenic, and causes alveolar echinococcosis (AE). The distribution of *E. multilocularis* is largely restricted to the Northern hemisphere and highest prevalence occurs in Central Asia, Russia, Northwestern China, and parts of Europe and Japan. Across the European endemic countries such as France, Germany, Austria and Switzerland, where AE cases were notified for many decades already in the past century, AE is basically still considered a rare disease with average incidences of 0.03–0.30/100,000 inhabitants/year [51]. These figures, however, do not reflect the situation of the actual population at risk; far higher incidences, from 4.7 to 8.1 cases per 100,000 inhabitants/year, are observed in areas where humans are directly exposed to *E. multilocularis* infection in the same countries [39, 47, 49]. In Europe and Canada, emergence and geographical increase in endemicity have been reported [7, 9, 16].

*Echinococcus granulosus sensu lato* (the small dog tapeworm) represents the most common species and occurs in both hemispheres; predominantly affected regions include the Mediterranean area, Eastern Europe, parts of South America, parts of Africa, and Central Asia/Western China.

Both parasites cause life-threatening diseases of serious public health and economic concern worldwide [58]. Mainly cystic echinococcosis (CE), but to some extent also alveolar echinococcosis (AE), are diseases of predominantly resource-poor low-income communities, but they also occur in middle-income or wealthy countries. For both CE and AE, the number of patients is most likely underestimated or underreported [27]. Globally, AE causes an annual loss of approximately 660,000 disability-adjusted life years [57]. Necessary long-term chemotherapy of echinococcosis using benzimidazoles, i.e. albendazole and mebendazole, which are the only available drugs with some efficacy, and the difficulty of making the decision for its safe withdrawal on clinical grounds are among the main factors for the economic cost of the disease, and the chronic impairment of patients’ quality of life [57, 59].

Epidemiologically, humans represent an aberrant “intermediate host” for these parasites. Infection is acquired through the accidental ingestion of eggs, released with the feces of infected carnivores, the “definitive hosts”. Eggs contain the infectious larval oncosphere, which actively penetrates the intestinal lining, and migrates via blood and lymphatic vessels to specific target sites. Most commonly affected organs in humans are the liver for *E. multilocularis* (AE), and the liver, lung, and many other sites for *E. granulosus* (CE). There, the oncosphere develops into the metacestode, with serious consequences for those individuals susceptible to its growth [28]. Within such a metacestode, protoscoleces might develop, which represent the infectious unit for definitive hosts. Development of protoscoleces depends on the host species and other, most likely immunological, factors provided by or lacking in their host. If such a fertile metacestode is ingested by a suitable definitive host, the life cycle is concluded, in that the protoscolex matures into an adult-stage tapeworm in the small intestine of this definitive host, and adult parasites start to produce parasite eggs 4 weeks p.i. for *E. multilocularis* and 5–6 weeks p.i. for *E. granulosus*.

Biologically, metacestodes are fluid-filled vesicles that are separated into (i) an inner “germinal layer” representing the living and metabolically active parasite tissue, and (ii) an outer, acellular compartment known as the “laminated layer”, mediating the direct physical contact with cells of the host’s inflammatory response, i.e. cells of the immune system and other cells such as parenchymal cells and fibroblasts. The distal part of the germinal layer, the tegument, is directly associated with the inner surface of the laminated layer. The tegument is characterized by microvilli-like extensions termed microtriches, which protrude well into the matrix of the laminated layer, and increase the resorbing surface of the parasite. In addition, the germinal layer contains highly differentiated cell types including connective tissue, muscle cells, and glycogen storage cells, as well as many undifferentiated cells, e.g. “stem” cells. The outer laminated layer is a carbohydrate-rich structure which is synthesized by the parasite and secreted into its peripheral entities, and which, in terms of thickness, is much more prominent in *E. granulosus* metacestodes where it is surrounded by a very prominent host-derived fibrous adventitial layer. In contrast to *E. granulosus*, *E. multilocularis* metacestodes are not surrounded by such an adventitial layer. Instead, the parasite larva represents a multi-vesicular organism that reproduces asexually, by exogenous formation of daughter vesicles. Periparasitic cells of the immune response exhibit a “granulomatous” arrangement and are directly in contact with the organ parenchyma on the one hand, and with the laminated layer of the budding daughter vesicles on the other. This process is often referred to as “progressive tumor-like growth”, and leads to the formation of a large and heterogeneous parasitic mass consisting of mostly peripheral, actively proliferating, sites, and in many cases, centrally located necrotic tissue. The release of metacestode structures such as microvesicles (for *E. multilocularis*) or protoscoleces (for *E. granulosus*) into the blood or lymphatic vessels may lead to metastases formation in other organs.

Spontaneous cure of AE or CE leading to calcified lesions is possible and even frequent, but it is not known how this event is initiated and terminated with such a favorable and fortunate prognosis. Mass screenings in endemic areas have revealed that the number of established AE infections in humans is far lower than the number of seropositive humans that had been exposed to the eggs of the parasite but remained clinically healthy, and following estimations, only 1–10% of individuals infected with *E. multilocularis* (as determined upon species-specific seropositivity) are assumed to subsequently develop disease (i.e. AE). Similarly, spontaneous abortion of already developed parasite cysts in *E. granulosus* infection, without any treatment, has been repeatedly shown by the teams that performed appropriate follow-up after mass screening in South America and China [15, 62].

This indicates that in the largest percentage of individuals, innate or acquired immunity can contribute to the control of the parasite for both species, either early or later after infection. Conversely, impairment of cellular immunity such as observed in advanced AIDS or other immunological disorders is associated with a rapid and unlimited growth and dissemination of the parasite in AE [9, 50]. CD4^+^ T-cell recovery in AIDS patients by means of appropriate therapy, however, reinstates control over progression of AE treated with benzimidazoles [69]. All these observations consequently open the way for development of appropriate immunotherapeutic tools, including vaccines, and for closer monitoring of patients in order to evaluate parasite viability, and thus to adjust and optimize anti-parasite therapies [66].

## 2. Viability: increase, decrease, dying-out

**Figure 1.**
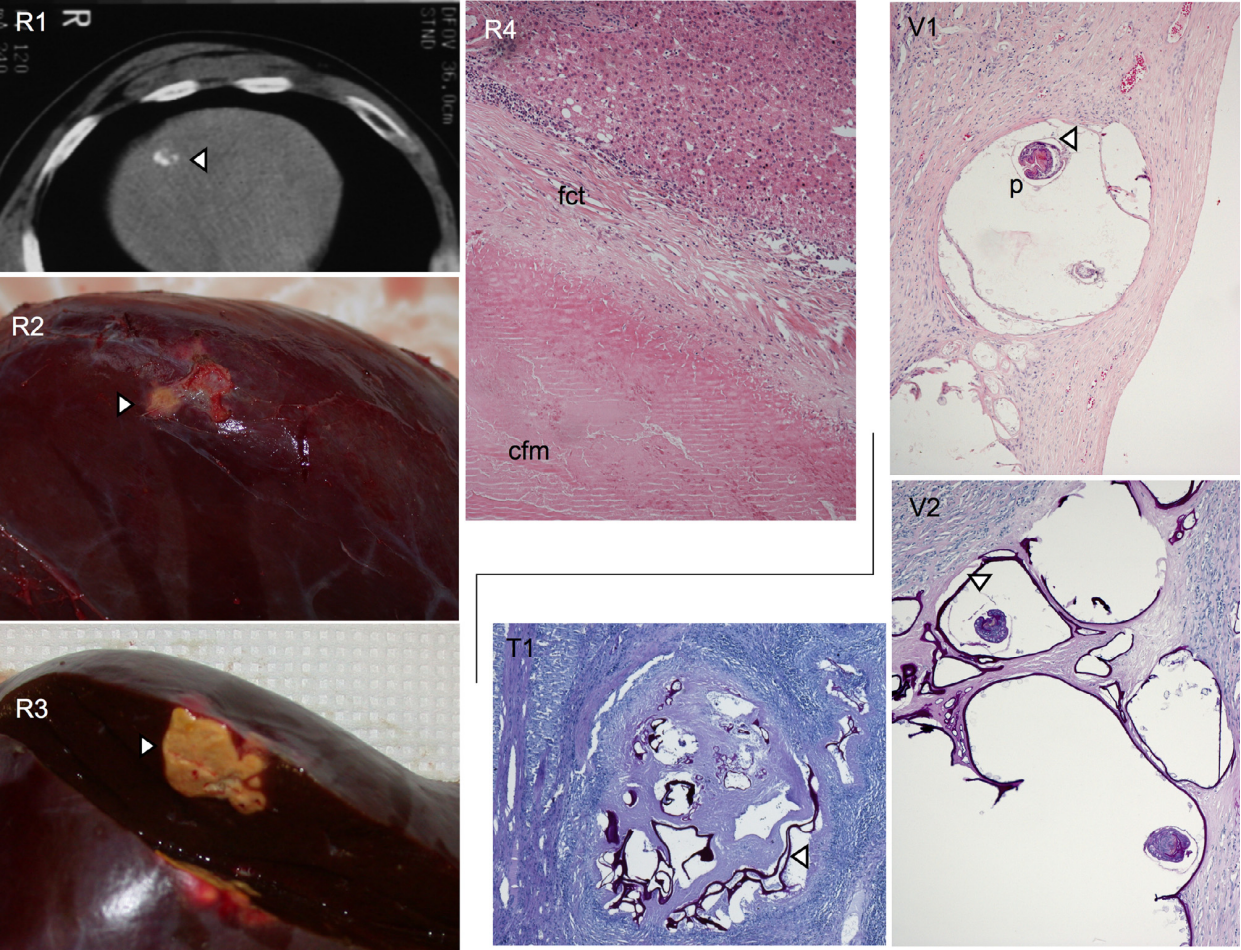
In addition to conventional AE found in human patients, so-called abortive (resistant, R) cases, where the parasite metacestode has spontaneously died out, are more and more frequently found. R1: by imaging techniques (e.g. CT), an abortive metacestode lesion (arrow) appears as a small, fully calcified structure. A surgically resected liver lobe containing such a lesion (arrows) is shown in R2 and following opening in R3. Histologically, an abortive lesion appears as an acellular structure, centrally composed of a collagenous and fibrous mass (cfm) void of any metacestode structures such as the germinal or laminated layers (R4, HE-stain; ×400); this mass is surrounded by a layer of fibrous connective tissue that still contains some laminated layer fibers and some parasite DNA (PCR-positivity of the material), but no remaining live parasite cells; there is still some inflammatory reaction around this died-out lesion, putatively responsible for the maintenance of a positive humoral immune response such as anti-Em2-seropositivity. In contrast, the classical viable AE metacestode lesion (V1, HE-stain; ×200) is composed of fluid-filled microvesicles that may rarely contain protoscolices (p); the actual living metacestode structure is the very thin germinal layer (arrow), which is closely adjacent to the PAS-positive outer laminated layer (V2, PAS-stain; ×200), indicated by the arrow. Before dying-out, the metacestode undergoes a transitional stage where it becomes more tightly encapsulated within a fibrous-collagenous mass, and where the germinal layers start to disappear, while the PAS-positive laminated layer still remains prominently present as vesiculated structures (T1, PAS-stain, ×100).

In infected humans, the *E. multilocularis* metacestode (larva) develops primarily in the liver, and a granulomatous host reaction surrounds the metacestode, including a vigorous synthesis of fibrous and healing tissue. In contrast to infections in susceptible rodent hosts, lesions from infected human patients rarely exhibit brood capsule and protoscolex formation within vesicles. The kind of immune response developed by the host accounts for the subsequent dichotomy concerning parasite development [54]: (i) resistance as shown by the presence of “dying-out” or “aborted” metacestodes (Figure 1); (ii) controlled susceptibility as shown by a slowly growing metacestode tissue – this group refers to normal AE patients who first experience clinical signs and symptoms 5–15 years after infection, and (iii) uncontrolled hyperproliferation of the metacestode due to an impaired immune response because of AIDS or other immunodeficiencies, e.g. following orthotopic liver transplantation, and in patients with drug-induced acquired immunodeficiency after other types of allogeneic transplantation or chemotherapy/biotherapy for malignant or chronic inflammatory diseases [9]. The host immune mechanisms modulating the course of infection include primarily T-cell interactions. Thus, the periparasitic granuloma contains a large number of CD4^+^ T cells in patients with abortive or dead lesions, whereas in patients with active metacestodes the number of CD8^+^ T cells is increased [61]. The parasitic metacestode itself seems to initiate, predominantly by means of bioactive metabolites, immunosuppressive and/or immunoregulatory processes that are assumed to correlate to parasite survival and proliferation dynamics [60]. Cytokine mRNA levels in peripheral blood mononuclear cells (PBMCs) during AE show initially elevated transcription levels of pro-inflammatory cytokines, e.g. IL-1β, IL-6 and tumor necrosis factor alpha (TNF-α), as well as Th1 cytokines, i.e. IL-12 and Interferon-γ, which are gradually re-oriented toward Th2, including elevated IL-13, IL-4, IL-5, and IL-10 [19, 61]. Transforming Growth Factor (TGF)-β transcripts are prominent in the periparasitic infiltrate which surrounds the lesions, both in the liver of patients with AE [65, 68] and in the liver of experimental mice [63]; in such mice, all members of the TGF-beta/Smad pathway are markedly expressed, and there is subtle interplay with members of the IL-17 family [63]. TGFβ-driven regulatory T cells were shown to play a crucial role in the parasite-modulated progressive course of AE [37]. However, among the immune cells which surround the metacestode, both Th1 and Th2 cytokine- and chemokine-secreting cells are numerous and IL-4 as well as INF-γ expression are increased from the very early stage of infection [63], which suggests that a secondary re-orientation of the immune response toward protection or tolerance may be crucial for the outcome of the infection. In the experimental murine model, such re-orientation toward resistance has been attributed to IL-12 [13], TNF-α [13, 25], and IFN-α [20]. In humans, only IFN-α administration was shown to lead toward some regression of AE lesions [22].

The response characteristics of AE-resistant persons have not been fully elucidated so far. One study suggested that, in patients with aborted lesions, secretion of IL-10 and Th-2 cytokines by PBMCs of AE patients was lower than in patients with a progressing disease [18]; another study suggested that progression of lesions, and the associated cytokine profile, could be related to genetic determinants of the immune response, such as the presence of the HLA-B8, DR3, DQ2 haplotype [17]. Other cytokines and chemokines secreted by the periparasitic immune cells also seem different in patients with regression *versus* progression of the disease [25, 29].

From the clinical point of view, *in vivo* assessment of metacestode activity status is essential with a view to designing an optimal individual treatment strategy for a given AE patient [6]. In addition, non-invasive methods would be preferred by the majority of clinicians who express remarkably strong demand for improved tools to assess *in vivo* the viability or non-viability status of treated hepatic and extra-hepatic echinococcosis lesions.

## 3. Imaging follow-up of AE and CE patients for parasite viability assessment

Progression of lesions, at least in immunocompetent patients, is extremely slow in AE, and prolonged follow-up is necessary before ruling on regression or progression. Improvement in the determination of lesion size has been proposed to improve the detection of changes in AE lesions [3]. However, whatever the imaging technique used, ultrasonography (US) [15], Magnetic Resonance Imaging (MRI), or Computed Tomography (CT), progression is better assessed than regression, and decrease in lesion size after drainage of a central cavity cannot be interpreted as regression; it simply corresponds to the shrinking of the central part of the lesion, irrespective of the viability of the peripheral metacestode vesicles [44]. Calcification of lesions, and its increase which indicates efficient protective immune response with subsequent degeneration of the parasite, cannot be used as a reliable marker of parasite death in the whole lesion [6]. Preliminary results from the comparison between MR images and [18F]-fluorodeoxyglucose (FDG) Positron Emission Tomography (PET) suggest that identification of microvesicles in AE lesions by MRI strongly suggests “metabolically active”, hence viable, metacestode [2]; however, before being reliably used as a marker, studies on a larger cohort of patients with and without anti-parasite treatment withdrawal will be necessary.

FDG is currently the only validated tracer of AE lesions; it was proposed 15 years ago to assess progression of lesions, if positive, and as a marker of parasite abortion and thus indication of benzimidazole withdrawal, if negative [43, 45]. This approach has proven effective in several patients, but drug withdrawal was followed by recurrence in some of them [45, 55]. Improvement in the PET imaging procedure, better adapted to the specific situation of AE, has been proposed: it is now accepted that PET may only be considered negative if images acquired 3 h (and not only 1 h) after FDG injection are negative [8]. In fact, FDG-PET images do not directly reflect parasite viability but rather periparasitic host inflammatory processes. This is supported by the location of FDG uptake by the lesion, only in those areas where the periparasitic infiltrate by immune cells is dense, and by an *in vitro* uptake of FDG by leucocytes far higher than the uptake by *E. multilocularis* cells or vesicles [42]. The correlation we found between images of microvesicles on MRI and positive FDG-PET is likely explained by more numerous and metabolically active immune cells in those areas of the lesions where active vesicles with clearly differentiated solid and liquid components are present [2].

The ideal PET tracer should be able to assess the course of AE upon direct uptake by the metacestode through its metabolic activity [41] or by binding to parasite molecules only present when the metacestode is viable. Respective candidates may be available among PET tracers already used for diagnosis or follow-up of other diseases such as [18F]-fluorotyrosine (FET), [18F]-fluorothymidine (FLT), [18F]-fluoromethylcholine (FMC), and sodium [18F]-fluorine (NaF) [41]. Preliminary *in vitro* results show that *E. multilocularis* germinal cells have a far higher uptake of Fluoro-L-Thymidine (FLT) than of FDG [42]. Such preclinical studies will require appropriate animal models for micro-PET/CT or MR imaging: mice and rats appear to be the most suitable animal species so far for susceptibility studies.

Therapeutically, one might consider using a similar tracer or designing ligand techniques making use of the specificity of anti-*Echinococcus* antibodies and of the cytotoxic properties of beta-radiation from radioisotopes, e.g. Lutetium 177 or Yttrium 90.

Ultrasonography (US) and CT have been assessed for evaluating efficacy of AE treatment [35]. Especially CT was suitable to classify the pattern of hepatic lesions into three types: solid form, pseudocystic form, and “geographic map” (mixed) form, which correlated well with other clinical findings that may either indicate cure or still progredient course of disease. Earlier reports [see 59] had already suggested that for an accurate disease evaluation, aiming to guide the surgical strategy, beside CT, Magnetic Resonance (MR) imaging, including cholangio-MR imaging can yield useful additional information.

The international classification of CE cysts has been designed with the purpose of differentiating between “active/progressing/viable cysts” and “inactive/degenerating/dead” cysts (ref WHO classification). CE1 and CE2 cysts are highly likely viable, and CE4 and CE5 cysts likely degenerating; CE3 cysts being considered to be “transitional cysts”, since they may either degenerate and move to CE4 or CE5, or give rise to daughter vesicles, as shown in CE2 [54]. Follow-up of patients, either in hospital settings or after mass screening, has however shown that CE1 cysts could spontaneously degenerate, and that CE4 and even CE5 cysts could sometimes show increase in size and progression [45, 53]. None of the available imaging procedures are currently able to accurately assess *E. granulosus* viability and/or predict cyst progression/abortion. Metabolic viability assessment of CE using high-field 1H-MRS of cyst content has been proposed to provide further information on cyst viability [24], but this approach has not yet received further validation. FDG-PET is negative in most cases of CE and is of no use for the assessment of parasite viability or disease progression. Nevertheless, through ultrasound, substantial progress has been made in recent years in understanding the natural and treatment-driven involution of CE cysts [54]. In addition, MRI reproduces US-defined features of CE better than CT. If US cannot be performed due to cyst location or patient-specific reasons, MRI with heavily T2-weighted series is preferable to CT [54]. As treatment decisions are driven by imaging findings, it is nevertheless important to know how US-based classification of CE cysts translates into MR- and CT-imaging for cases where MRI and CT substitute for US. This holds especially true for cysts that are not accessible to US. Larrieu et al. [30] have successfully used ultrasonography to monitor treatment of CE with albendazole. Ultrasonography was also used to understand how cyst structures alter with time to evaluate the effectiveness of different treatment regimes and to understand natural degeneration of cysts that can occur. Such studies have been undertaken in various geographically and ethnically diverse communities [11, 34, 47]. Ultrasonography, for the same purposes, also proved useful for pediatric CE cases [17].

## 4. Biological follow-up for CE patients

Any kind of treatment for any case of CE, including surgery, implies long-term follow-up of the patient, which is needed to detect relapses. Based on the standardized US-based monitoring used for the follow-up of CE patients, for some specific cases, cyst puncture with direct assessment of parasite viability (assessment of the presence of protoscoleces) may be proposed. However, this type of invasive technique cannot be recommended and hardly applies to most of the patients treated by benzimidazoles for multiple cysts and/or cysts in locations inaccessible to safe puncture.

In parallel, follow-up laboratory analyses to monitor drug tolerance include liver enzymes (aminotransferases) and leukocyte count; they provide little information on the sensitivity of the parasite to treatment. The search for immunological parameters useful in the staging and prognosis of CE in treated patients has yielded scarce practical tools so far. Presence of circulating antibodies against parasite antigens is observed in only 50–80% of patients, depending on the stage and location of the cyst(s) [10, 14, 21], and most of the target antigens for the antibodies that are the most efficient for diagnosis remain present in cysts which have lost any viability, which prevents the use of the corresponding antibodies for follow-up. In those patients with positive serology at diagnosis, follow-up of antibody isotypes and immune complexes was already proposed a long time ago to discriminate between responders and non-responders to albendazole treatment. However, since then, the usefulness of immune-complexes monitoring has not been validated by other teams. Siracusano et al. [53] reported on HSP20 as a potential marker of active CE. Their immunoblot analysis revealed anti-HSP20 antibodies in a statistically significant higher percentage of sera from patients with active disease than in sera from patients with inactive disease. Anti-HSP20 antibody levels significantly decreased over the course of chemotherapy in sera from patients with cured disease, when compared to sera from patients with progressive disease. Hernandez-Gonzalez et al. [23] showed that the B2t-IgG-ELISA is of a better prognostic value for the follow-up of surgically treated CE patients than other conventional *Echinococcus*-ELISAs. Similar findings were reported by Ben Nouir et al. [4], who documented a relatively rapid decrease of anti-recP29 antibody levels in young CE patients who had been successfully treated by surgery, while relapsing CE patients continued or newly developed an anti-recP29 level following treatment. Post-treatment positive specific IgG antibody seroconversion against recAgB in initially seronegative CE1 patients was considered a good indication for positive therapeutic efficacy of albendazole, as reported by Li et al. [33]. Prospectively, promising new tools helpful for post-treatment monitoring of CE patients by laboratory methods may rather be developed in the domain of circulating antigen detection [14], the disappearance/absence of which should more rapidly reflect parasite inactivation than conventional antibody detection [67]. A recent study showed that circulating antigen B was present in the sera of 90% of antibody-negative CE patients and suggested that its detection could markedly improve CE diagnosis [32]; however, circulating antigens and/or their levels have not been fully studied as viability markers as yet [48].

A correlation between CE stage and selected Th1 versus Th2 cytokine profiles was investigated by Piccoli et al. [40]. With the exception of the association of IL-4 and IL-13 with the cyst stage, all other cytokines did not associate with cyst stages. A more optimistic presentation of using cytokines to assess efficacy of CE treatment was reviewed by Siracusano et al. [53]. Their conclusion was that patients who responded to chemotherapy produced high amounts of IFN-γ, whereas patients who did not respond produced IL-4 and IL-10. At the mRNA level, patients in whom therapy failed, weakly expressed IL-4 mRNA before therapy, and strongly thereafter; patients who responded to therapy expressed higher IFN-γ and TNF-α mRNA values than patients who did not [46].

## 5. Biological follow-up for AE patients

In AE patients, testing of parasite viability can be performed with RT-PCR of biopsies and fine-needle aspirates on various constitutively expressed gene targets (e.g. 14-3-3) [12], within the limits of the sensitivity of this method [26, 36, 65]. However, due to these limits, this invasive technique cannot yet be recommended for routine follow-up.

In AE patients, follow-up after surgery and chemotherapy is probably best life-long. Cases of recurrence have been observed almost 20 years after surgery [1]. Better than for CE, in AE some specific serologic tests are valuable to assess the efficacy of treatment in combination with imaging during follow-up of patients [26, 52]. Disappearance of IgE and IgG4 isotypes of anti-*Echinococcus* specific antibodies was reported to be associated with good prognosis and absence of recurrence after surgery and chemotherapy in AE patients; conversely, reappearance of specific IgG4 antibodies was a strong indication of recurrence [67]. This was confirmed in another series of patients, which also showed that in AE patients with progressive disease, IgG4 distinctively recognized low molecular weight EmAg of Mr 26 kDa, 18 kDa, 16 kDa, and 12 kDa [reviewed in 52]. In a more recent study, *E. multilocularis* metacestode-specific IgG1, IgG3, and IgE responses progressively diminished with regression from active to stable and cured AE. IgG2 and IgG4 reactivity remained similarly high in stable and progressive cases, and lessened only with cured AE [25]. However, such isotype determinations do not seem to have entered routine follow-up of AE patients, and should perhaps be evaluated further in a larger series of patients.

After successful surgery and/or chemotherapy leading to inactivation of the parasite, anti-Em18 (and to a certain extent anti-Em2^+^) antibody decline is rapid, and seroconversion to undetectable levels correlates well with curative resection [1, 5, 56]. It is likely that this type of antibody detection for follow-up will rely on thresholds different from those established for diagnosis [5]; definition of “follow-up thresholds” as well as the value of combined antibody and FDG-PET images need to be assessed in a significant number of patients with prospective follow-up. As suggested above for CE, for post-treatment monitoring of AE patients, the detection of circulating antigens may more directly reflect parasite metabolism and thus viability, or, in case of absence, parasite inactivation. Presence and, if positive, concentrations of circulating *E. multilocularis* DNA could also be studied in patients with active lesions before and after various types of treatment.

Cytokine and chemokine levels, alone or combined with other circulating markers, might be used as follow-up markers: in a recent study of German patients with AE, the spontaneous cellular release of pro-inflammatory IL-31 and IL-33 was clearly depressed in all AE patients, while regulatory IL-27, anti-inflammatory SDF-1/CXCL12, and eosinophil granulocyte attracting Eotaxin-1, Eotaxin-2, and Eotaxin-3 (CCL11, CCL24, CCL26) were enhanced with disease progression [25].

Kocherscheidt et al. [29] compared characteristic chemokine profiles and changes in their production in AE patients versus infection-free controls in antigen-stimulated PBMCs. The production of CC and CXC chemokines that associate with inflammation (MIP-1 alpha/CCL3, MIP-1 beta/CCL4, RANTES/CCL5, and GRO-alpha/CXCL1) was constitutively larger in AE patients than in controls; and the elevated chemokine releases were equal in patients with progressive, stable, or cured AE. A disparate cellular responsiveness was observed in AE patients upon chemokine cluster analyses: cluster 1 and cluster 2 chemokines were clearly diminished, while cluster 3 chemokines augmented in AE patients. Lechner et al. [31] studied the pro-inflammatory IL-17 cytokine family members and their common receptors. The study disclosed divergent cellular production profiles and plasma levels in AE patient groups and controls; Th17-type IL-17A levels were similar in patients with progressive, stable, and cured *E. multilocularis* metacestode lesions, IL-17B increased in AE patients, while the Th17-type IL-17F production was highest in controls and depressed in all AE patient groups similarly [31].

The regulatory T-cell effector Fibrinogen-Like Protein 2 (FGL2) is a recent additional candidate to discriminate between patients with active and inactive lesions: its role as an important mediator of the tolerance status in *E. multilocularis* infection has received experimental confirmation [64]; preliminary results have shown that its soluble form is significantly increased in the serum of patients with AE compared to healthy subjects; however, its association with disease course and prognosis is still under investigation (Junhua Wang, personal communication).

## 6. Conclusions

For both CE and AE, clinicians have to assess efficacy and efficiency of treatment protocols, and to decide whether treatment has reached an endpoint where the parasite has lost all potential for further proliferation and thus where the disease is definitively extinct. The search for improved possibilities to determine metacestode viability *in vivo* is ongoing. There is an urgent need for well-validated non-invasive markers: they may consist of imaging features or biological markers or both. The significant advances in our knowledge of *Echinococcus* biology at the molecular level and on the host’s immune response have now facilitated the search for novel and potentially suitable follow-up tools that will provide the sufficient clinical information needed to reliably stop anti-parasite treatment. Validation of new tools, however, relies on the capacity of clinical research teams to set up patient cohorts that will make statistical comparisons relevant and enable recommendations based on solid evidence. To reach this goal, it is thus important to increase public awareness of the impact of AE and CE on society, in order to generate resources to carry out such research and studies.

## References

[1] Ammann RW, Renner EC, Gottstein B, Grimm F, Eckert J, Renner EL, Swiss Echinococcosis Study Group. 2004 Immunosurveillance of alveolar echinococcosis by specific humoral and cellular immune tests: prospective long-term analysis of the Swiss chemotherapy trial (1976–2001). Journal of Hepatology, 41, 551–559. 15532108 10.1016/j.jhep.2004.06.015

[2] Azizi A, Blagosklonov O, Ahmed L, Berthet L, Vuitton DA, Bresson-Hadni S, Delabrousse E. 2014 Alveolar echinococcosis: correlation between MRI aspect of hepatic lesions and the metabolic activity visualized in FDG-PET/CT. In: Proceedings of the International Symposium “Innovation for the Management of Echinococcosis”, Besançon, March 27–29, 2014, p. 10.

[3] Becce F, Pomoni A, Uldry E, Halkic N, Yan P, Meuli R, Schmidt S. 2014 Alveolar echinococcosis of the liver: diffusion-weighted MRI findings and potential role in lesion characterisation. European Journal of Radiology, 83, 625–631. 24457140 10.1016/j.ejrad.2013.12.025

[4] Ben Nouir N, Gianinazzi C, Gorcii M, Müller N, Nouri A, Babba H, Gottstein B. 2009 Assessment of *Echinococcus granulosus* somatic protoscolex antigens for serological follow-up of young patients surgically treated for cystic echinococcosis. Transactions of the Royal Society of Tropical Medicine and Hygiene, 103, 355–364. 19027129 10.1016/j.trstmh.2008.09.020

[5] Bresson-Hadni S, Blagosklonov O, Knapp J, Grenouillet F, Sako Y, Delabrousse E, Brientini MP, Richou C, Minello A, Antonino AT, Ito A, Mantion GA, Vuitton DA. 2011 Should possible recurrence of disease contraindicate liver transplantation in patients with end-stage alveolar echinococcosis? A 20-year followup study. Liver Transplantation, 17, 855–865. 21455928 10.1002/lt.22299

[6] Brunetti E, Kern P, Vuitton DA, Writing Panel for the WHO-IWGE. 2010 Expert consensus for the diagnosis and treatment of cystic and alveolar echinococcosis in humans. Acta Tropica, 114, 1–16. 19931502 10.1016/j.actatropica.2009.11.001

[7] Bruzinskaite R, Marcinkute A, Strupas K, Sokolovas V, Deplazes P, Mathis A, Eddi C, Sarkūnas M. 2007 Alveolar Echinococcosis, Lithuania. Emerging Infectious Diseases, 13, 1618–1619. 18258025 10.3201/eid1310.061161 PMC2851530

[8] Caoduro C, Porot C, Vuitton DA, Bresson-Hadni S, Grenouillet F, Richou C, Boulahdour H, Blagosklonov O. 2013 The role of delayed 18F-FDG PET imaging in the follow-up of patients with alveolar echinococcosis. Journal of Nuclear Medicine, 54, 358–363. 23303963 10.2967/jnumed.112.109942

[9] Chauchet A, Grenouillet F, Knapp J, Richou C, Delabrousse E, Dentan C, Capelle S, Di Martino V, Deconinck E, Blagosklonov O, Vuitton DA, Bresson-Handi S. 2013 Emergence of a new opportunistic infection in Europe: hepatic alveolar echinococcosis. Fifty case-report. Journal of Hepatology, 58 (Suppl 11), S381.

[10] Craig PS. 1986 Detection of specific circulating antigen, immune complexes and antibodies in human hydatidosis from Turkana (Kenya) and Great Britain, by enzyme-immunoassay. Parasite Immunology, 8, 171–188. 3517766 10.1111/j.1365-3024.1986.tb00843.x

[11] Del Carpio M, Mercapide CH, Salvitti JC, Uchiumi L, Sustercic J, Panomarenko H, Moguilensky J, Herrero E, Talmon G, Volpe M, Araya D, Mujica G, Calabro A, Mancini S, Chiosso C, Labanchi JL, Saad R, Goblirsch S, Brunetti E, Larrieu E. 2012 Early diagnosis, treatment and follow-up of cystic echinococcosis in remote rural areas in Patagonia: impact of ultrasound training of non-specialists. PLoS Neglected Tropical Diseases, 6(1), e1444. 22253935 10.1371/journal.pntd.0001444 PMC3254659

[12] Diebold Berger S, Khan H, Gottstein B, Puget E, Frossard JL, Remadi S. 1997 Cytologic diagnosis of isolated pancreatic alveolar hydatid disease with immunologic and PCR analyses – a case report. Acta Cytologica, 41, 1381–1386. 9990280 10.1159/000333544

[13] Emery I, Leclerc C, Sengphommachanh K, Vuitton DA, Liance M. 1998 In vivo treatment with recombinant IL-12 protects C57BL/6J mice against secondary alveolar echinococcosis. Parasite Immunology, 20, 81–91. 9572051 10.1046/j.1365-3024.1998.00131.x

[14] Ferragut G, Ljungström I, Nieto A. 1998 Relevance of circulating antigen detection to follow-up experimental and human cystic hydatid infections. Parasite Immunology, 20, 541–549. 9988311 10.1046/j.1365-3024.1998.00177.x

[15] Frider B, Moguilensky J, Salvitti JC, Odriozola M, Cantoni G, Larrieu E. 2001 Epidemiological surveillance of human hydatidosis by means of ultrasonography: its contribution to the evaluation of control programs. Acta Tropica, 79, 219–223. 11412805 10.1016/s0001-706x(01)00096-1

[16] Gesy K, Hill JE, Schwantje H, Liccioli S, Jenkins EJ. 2013 Establishment of a European-type strain of *Echinococcus multilocularis* in Canadian wildlife. Parasitology, 140, 1133–1137. 23714582 10.1017/S0031182013000607

[17] Gocan H, Surd A, Dobrescu I, Pop E. 2010 The role of ultrasonography in Albendazole treatment of hydatid liver cyst monitoring in children – three case reports. Medical Ultrasonography Journal, 12, 340–344. 21210021

[18] Godot V, Harraga S, Beurton I, Deschaseaux M, Sarciron E, Gottstein B, Vuitton DA. 2000 Resistance/susceptibility to *Echinococcus multilocularis* infection and cytokine profile in humans. I. Comparison of patients with progressive and abortive lesions. Clinical and Experimental Immunology, 121, 484–490. 10971515 10.1046/j.1365-2249.2000.01308.x PMC1905721

[19] Godot V, Harraga S, Beurton I, Tiberghien P, Sarciron E, Gottstein B, Vuitton DA. 2000 Resistance/susceptibility to *Echinococcus multilocularis* infection and cytokine profile in humans. II. Influence of the HLA B8, DR3, DQ2 haplotype. Clinical and Experimental Immunology, 121, 491–498. 10971516 10.1046/j.1365-2249.2000.01309.x PMC1905739

[20] Godot V, Harraga S, Podoprigora G, Liance M, Bardonnet K, Vuitton DA. 2003 IFN alpha-2a protects mice against a helminth infection of the liver and modulates immune responses. Gastroenterology, 124, 1441–1450. 12730883 10.1016/s0016-5085(03)00273-7

[21] Gottstein B. 1984 An immunoassay for the detection of circulating antigens in human echinococcosis. American Journal of Tropical Medicine and Hygiene, 33, 1185–1191. 6391225 10.4269/ajtmh.1984.33.1185

[22] Harraga S, Godot V, Bresson-Hadni S, Pater C, Beurton I, Bartholomot B, Vuitton DA. 1999 Clinical efficacy of and switch from T helper 2 to T helper 1 cytokine profile after interferon alpha2a monotherapy for human echinococcosis. Clinical Infectious Diseases, 29, 205–206. 10433588 10.1086/520157

[23] Hernández-González A, Muro A, Barrera I, Ramos G, Orduña A, Siles-Lucas M. 2008 Usefulness of Four different *Echinococcus granulosus* recombinant antigens for serodiagnosis of unilocular hydatid disease (UHD) and postsurgical follow-up of patients treated for UHD. Clinical Vaccine Immunology, 15, 147–153. 17989342 10.1128/CVI.00363-07 PMC2223859

[24] Hosch W, Stojkovic M, Jänisch T, Kauffmann GW, Junghanss T. 2007 The role of calcification for staging cystic echinococcosis (CE). European Radiology, 17, 2538–2545. 17473925 10.1007/s00330-007-0638-6

[25] Huang X, Grüner B, Lechner CJ, Kern P, Soboslay PT. 2014 Distinctive cytokine, chemokine, and antibody responses in *Echinococcus multilocularis*-infected patients with cured, stable, or progressive disease. Med Microbiological Immunology, 203, 185–193. 10.1007/s00430-014-0331-8 24509604

[26] Ito A, Craig PS. 2003 Immunodiagnostic and molecular approaches for the detection of taeniid cestode infections. Trends in Parasitology, 19, 377–381. 12957509 10.1016/s1471-4922(03)00200-9

[27] Jorgensen P, van der Heiden M, Kern P, Schöneberg I, Krause G, Alpers K. 2008 Underreporting of human alveolar echinococcosis, Germany. Emerging Infectious Diseases, 14, 935–937. 18507906 10.3201/eid1406.071173 PMC2600310

[28] Kern P. 2010 Clinical features and treatment of alveolar echinococcosis. Current Opinion in Infectious Diseases, 23, 505–512. 20683265 10.1097/QCO.0b013e32833d7516

[29] Kocherscheidt L, Flakowski AK, Grüner B, Hamm DM, Dietz K, Kern P, Soboslay PT. 2008 *Echinococcus multilocularis*: inflammatory and regulatory chemokine responses in patients with progressive, stable and cured alveolar echinococcosis. Experimental Parasitology, 119, 467–474. 18490012 10.1016/j.exppara.2008.04.006

[30] Larrieu E, Del Carpio M, Mercapide CH, Salvitti JC, Sustercic J, Moguilensky J, Panomarenko H, Uchiumi L, Herrero E, Talmon G, Volpe M, Araya D, Mujica G, Mancini S, Labanchi JL, Odriozola M. 2011 Programme for ultrasound diagnoses and treatment with albendazole of cystic echinococcosis in asymptomatic carriers: 10 years of follow-up of cases. Acta Tropica, 117, 1–5. 20832386 10.1016/j.actatropica.2010.08.006

[31] Lechner C, Grüner B, Huang X, Hoffmann WH, Kern P, Soboslay PT. 2012 Parasite-specific IL-17-type cytokine responses and soluble IL-17 receptor levels in alveolar echinococcosis patients. Clinical and Developmental Immunology, Article ID: 735342. 10.1155/2012/735342 PMC3437316 22969818

[32] Li J, Zhang WB, Lin RY, Wang H, Li L, Wang JH, McManus DP, Wen H. 2014 Circulating Antigen B in Cystic Echinococcosis patients antibody-negative against hydatid cyst fluid antigens. Parasite (in press).

[33] Li T, Ito A, Pengcuo R, Sako Y, Chen X, Qiu D, Xiao N, Craig PS. 2011 Post-treatment follow-up study of abdominal cystic echinococcosis in Tibetan communities of Northwest Sichuan province, China. PLoS Neglected Tropical Diseases, 5, e1364. 22039558 10.1371/journal.pntd.0001364 PMC3201905

[34] Li T, Ito A, Pengcuo R, Sako Y, Chen X, Qiu D, Xiao N, Craig PS. 2011 Post-treatment follow-up study of abdominal cystic echinococcosis in Tibetan communities of northwest Sichuan Province, China. PloS Neglected Tropical Diseases, 5(10), e1364. 22039558 10.1371/journal.pntd.0001364 PMC3201905

[35] Liu YH, Wang XG, Gao JS, Qingyao Y, Horton J. 2009 Continuous albendazole therapy in alveolar echinococcosis: long-term follow-up observation of 20 cases. Transactions of the Royal Society of Tropical Medicine and Hygiene, 103, 768–778. 19457528 10.1016/j.trstmh.2009.04.006

[36] Matsumoto J, Müller N, Hemphill A, Oku Y, Kamiya M, Gottstein B. 2006 14–3-3- and II/3-10-gene expression as molecular markers to address viability and growth activity of *Echinococcus multilocularis* metacestode. Parasitology, 132, 83–94. 16393357 10.1017/S0031182005008632

[37] Mejri N, Mueller J, Gottstein B. 2011 Intraperitoneal murine *Echinococcus multilocularis* infection induces differentiation of TGF-β expressing DCs that remain immature. Parasite Immunology, 33, 471–482. 21609335 10.1111/j.1365-3024.2011.01303.x

[38] Nakao M, McManus DP, Schantz PM, Craig PS, Ito A. 2007 A molecular phylogeny of the genus *Echinococcus* inferred from complete mitochondrial genomes. Parasitology, 134, 713–722. 17156584 10.1017/S0031182006001934

[39] Piarroux M, Piarroux R, Giorgi R, Knapp J, Bardonnet K, Sudre B, Watelet J, Dumortier J, Gérard A, Beytout J, Abergel A, Mantion G, Vuitton DA, Bresson-Hadni S. 2013 Populations at risk for alveolar echinococcosis, France. Emerging Infectious Diseases, 19, 721–728. 23647623 10.3201/eid1905.120867 PMC3647496

[40] Piccoli L, Meroni V, Genco F, Tamarozzi F, Tinelli C, Filice C, Brunetti E. 2012 Serum cytokine profile by ELISA in patients with echinococcal cysts of the liver: a stage-specific approach to assess their biological activity. Clinical and Developmental Immunology, 2012, 483935. 22400036 10.1155/2012/483935 PMC3287044

[41] Porot C, Wang J, Germain S, Seimbille Y, Camporese D, Knapp J, Vuitton DA, Blagosklonov O, Gottstein B. 2013 In vitro and in vivo investigations to develop functional imaging by Positron Emission Tomography (PET) for murine and human alveolar echinococcosis, Abstract book, 24th WAAVP Meeting, Perth, Australia.

[42] Porot C, Knapp J, Wang J, Camporese D, Germain S, Seimbille Y, Vuitton DA, Gottstein B, Blagosklonov O. 2014 Development of a Specific tracer for alveolar echinococcosis metabolic imaging: a preclinical study. Proc. IEEE Eng. Med. Biol. Soc. (in press). 10.1109/EMBC.2014.6944893 25571261

[43] Reuter S, Schirrmeister H, Kratzer W, Dreweck C, Reske SN, Kern P. 1999 Pericystic metabolic activity in alveolar echinococcosis: assessment and follow-up by positron emission tomography. Clinical Infectious Diseases, 29, 1157–1163. 10524957 10.1086/313438

[44] Reuter S, Nüssle K, Kolokythas O, Haug U, Rieber A, Kern P, Kratzer W. 2001 Alveolar liver echinococcosis: a comparative study of three imaging techniques. Infection, 29, 119–125. 11440381 10.1007/s15010-001-1081-2

[45] Reuter S, Buck A, Manfras B, Kratzer W, Seitz HM, Darge K, Reske SN, Kern P. 2004 Structured treatment interruption in patients with alveolar echinococcosis. Hepatology, 39, 509–517. 14768005 10.1002/hep.20078

[46] Riganò R, Profumo E, Buttari B, Teggi A, Siracusano A. 1999 Cytokine gene expression in peripheral blood mononuclear cells (PBMC) from patients with pharmacologically treated cystic echinococcosis. Clinical and Experimental Immunology, 118, 95–101. 10540165 10.1046/j.1365-2249.1999.01021.x PMC1905393

[47] Rogan MT, Hai WY, Richardson R, Zeyhle E, Craig PS. 2006 Hydatid cysts: Does every picture tell a story? Trends in Parasitology, 22, 431–438. 16843726 10.1016/j.pt.2006.07.003

[48] Rouhani S, Parvizi P, Spotin A. 2013 Using specific synthetic peptide (p176) derived AgB 8/1-kDa accompanied by modified patient’s sera: a novel hypothesis to follow-up of Cystic echinococcosis after surgery. Medical Hypotheses, 81, 557–560. 23890801 10.1016/j.mehy.2013.07.003

[49] Said-Ali Z, Grenouillet F, Knapp J, Bresson-Hadni S, Vuitton DA, Raoul F, Richou C, Millon L, Giraudoux P, Francechino Network. 2013 Detecting nested clusters of human alveolar echinococcosis. Parasitology, 140, 1693–1700. 23962413 10.1017/S0031182013001352

[50] Sailer M, Soelder B, Allerberger F, Zaknun D, Feichtinger H, Gottstein B. 1997 Alveolar echinococcosis of the liver in a six-year-old girl with acquired immunodeficiency syndrome. Journal of Pediatrics, 130, 320–323. 9042141 10.1016/s0022-3476(97)70364-0

[51] Schweiger A, Ammann RW, Candinas D, Clavien PA, Eckert J, Gottstein B, Halkic N, Muellhaupt B, Prinz BM, Reichen J, Tarr PE, Torgerson PR, Deplazes P. 2007 Human alveolar echinococcosis after fox population increase, Switzerland. Emerging Infectious Diseases, 13, 878–882. 17553227 10.3201/eid1306.061074 PMC2792858

[52] Siles-Lucas S, Gottstein B. 2001 Review: molecular tools for the diagnosis of cystic and alveolar echinococcosis. Tropical Medicine and International Health, 6, 463–475. 11422961 10.1046/j.1365-3156.2001.00732.x

[53] Siracusano A, Delunardo F, Teggi A, Ortona E. 2012 Host-Parasite relationship in cystic echinococcosis: an evolving story. Clinical and Developmental Immunology, Article ID: 639362. 10.1155/2012/639362 PMC3206507 22110535

[54] Stojkovic M, Gottstein B, Junghanns T. 2014 Echinococcosis, in Manson’s Tropical Diseases, 23rd edn., Farrar J, Hotez PJ, Junghanss T, Kang G, Lalloo D, White N, Editors. Elsevier Saunders: Amsterdam p. 795–819.

[55] Stumpe KD, Renner-Schneiter EC, Kuenzle AK, Grimm F, Kadry Z, Clavien PA, Deplazes P, von Schulthess GK, Muellhaupt B, Ammann RW, Renner EL. 2007 F-18-fluorodeoxyglucose (FDG) positron-emission tomography of *Echinococcus multilocularis* liver lesions: prospective evaluation of its value for diagnosis and follow-up during benzimidazole therapy. Infection, 35, 11–18. 17297583 10.1007/s15010-007-6133-9

[56] Tappe D, Sako Y, Itoh S, Frosch M, Grüner B, Kern P, Ito A. 2010 Immunoglobulin G subclass responses to recombinant Em18 in the follow-up of patients with alveolar echinococcosis in different clinical stages. Clinical Vaccine Immunology, 17, 944–948. 20392888 10.1128/CVI.00026-10 PMC2884418

[57] Torgerson PR, Schweiger A, Deplazes P, Pohar M, Reichen J, Ammann RW, Tarr PE, Halkik N, Müllhaupt B. 2008 Alveolar echinococcosis: from a deadly disease to a well-controlled infection. Relative survival and economic analysis in Switzerland over the last 35 years. Journal of Hepatology, 49, 72. 18485517 10.1016/j.jhep.2008.03.023

[58] Torgerson PR. 2003 Economic effects of echinococcosis. Acta Tropica, 85, 113–118. 12606088 10.1016/s0001-706x(02)00228-0

[59] Vuitton DA, Bresson-Hadni S. 2014 Alveolar echinococcosis: evaluation of the therapeutic strategies. Expert Opinion on Orphan Drugs, 2, 67–86.

[60] Vuitton DA, Gottstein B. 2010 *Echinococcus multilocularis* and its intermediate host: a model of parasite-host interplay. Journal of Biomedicine and Biotechnology, 2010, 923193. 20339517 10.1155/2010/923193 PMC2842905

[61] Vuitton D, Zhang SL, Yang Y, Godot V, Beurton I, Mantion G, Bresson-Hadni S. 2006 Survival strategy of *Echinococcus multilocularis* in the human host. Parasitology International, Suppl., 55, S51–S55. 16360335 10.1016/j.parint.2005.11.007

[62] Wang L, Wen H, Feng X, Jiang X, Duan X. 2012 Analysis of economic burden for patients with cystic echinococcosis in five hospitals in northwest China. Transactions of the Royal Society of Tropical Medicine and Hygiene, 106, 743–748. 23122883 10.1016/j.trstmh.2012.09.003

[63] Wang J, Lin R, Zhang W, Li L, Gottstein B, Blagosklonov O, Lü G, Zhang C, Lu X, Vuitton DA, Wen H. 2014 Transcriptional profiles of cytokine/chemokine factors of immune cell-homing to the parasitic lesions: a comprehensive one-year course study in the liver of *E. multilocularis*-infected mice. PLoS One, 9, e91638. 24637903 10.1371/journal.pone.0091638 PMC3956718

[64] Wang J, Vuitton DA, Müller N, Blagosklonov O, Grandgirard D, Leib SL, Shalev I, Levy G, Lu X, Lin R, Wen H, Spiliotis M, Gottstein B. submitted. Deletion of fibrinogen-like protein 2 (FGL-2), a novel CD4+ CD25+ Treg effector molecule, leads to improved control of *Echinococcus mutilocularis* infection. European Journal of Immunology. 10.1371/journal.pntd.0003755 PMC4425495 25955764

[65] Yamasaki H, Nakaya K, Nakao M, Sako Y, Ito Akira. 2007 Significance of molecular diagnosis using histopathological specimens in cestode zoonoses. Tropical Medicine and Health, 35, 307–321.

[66] Zhang W, Li L, McManus DP. 2003 Concepts in immunology and diagnosis of hydatid disease. Clinical Microbiology Review, 16, 16–36. 10.1128/CMR.16.1.18-36.2003 PMC145297 12525423

[67] Zhang W, Wen H, Li J, Lin R, McManus DP. 2012 Immunology and immunodiagnosis of cystic echinococcosis: an update. Clinical and Developmental Immunology, Article ID: 101895, doi: 10.1155/2012/101895. 10.1155/2012/101895 PMC3253442 22235225

[68] Zhang S, Hüe S, Sène D, Penfornis A, Bresson-Hadni S, Kantelip B, Caillat-Zucman S, Vuitton DA. 2008 Expression of major histocompatibility complex class I chain-related molecule A, NKG2D, and transforming growth factor-beta in the liver of humans with alveolar echinococcosis: new actors in the tolerance to parasites? Journal of Infectious Diseases, 197, 1341–1349. 18422447 10.1086/586709

[69] Zingg W, Renner-Schneiter EC, Pauli-Magnus C, Renner EL, van Overbeck J, Schläpfer E, Weber M, Weber R, Opravil M, Gottstein B, Speck RF, the Swiss HIV Cohort Study. 2004 Alveolar echinococcosis of the liver in an adult with human immunodeficiency virus type-1 infection. Infection, 32, 299–302. 15624896 10.1007/s15010-004-3134-9

